# Physicochemical Properties of Moderately Heat-Treated Rice Protein Within Alkaline Solution and Its Evaluation as a Spray-Drying Microencapsulation Wall Material

**DOI:** 10.3390/foods14101739

**Published:** 2025-05-14

**Authors:** Mengqi Liu, Rumeng Huang, Lifeng Wang, Mohamed Eid, Wenfei Xiong

**Affiliations:** 1 College of Food Science and Engineering/Collaborative Innovation Center for Modern Grain Circulation and Safety, Nanjing University of Finance and Economics, Nanjing 210023, China; 2 Department of Biochemistry, Faculty of Agriculture, Benha University, Moshtohor 13736, Qaliuobia, Egypt; mohamed.eed@fagr.bu.edu.eg; 3 Jiangsu Key Laboratory of Advanced Food Manufacturing Equipment & Technology, Jiangnan University, Wuxi 214122, China

**Keywords:** rice glutelin, depolymerization, subunit degradation, emulsification properties, redispersibility

## Abstract

This study addresses the shortcoming of rice protein, which has limited its widespread use as a food ingredient due to its extremely low solubility in neutral aqueous solution. Herein, rice protein (RP) was dispersed in aqueous solutions with different alkali concentrations (0.075 M~0.125 M), and then heat-treated (80 °C, 1~4 h) to obtain a modified RP. The physicochemical properties of the modified RP in neutral aqueous solution and its performance as a microencapsulated wall material were then comprehensively analyzed. The results showed that the solubility of the RP at pH 7.0 could be increased to more than 56.3% by alkali solution combined with moderate heat treatment for 1 h. Further analysis revealed that the enhancement of the RP solubility performance was mainly due to the depolymerization of rice glutenin cluster aggregates, with the average size decreasing to 140~180 nm, which was also accompanied by an increase in net zeta potential. Structural analysis pointed to a significant decrease in the surface hydrophobicity and free sulfhydryl content of the RP after thermal treatment in alkaline solution, while degradation of glutenin subunits (especially for the results of alkaline treatment at higher concentrations) and an increase in random coil content occurred. These physicochemical properties and conformational transitions of the modified RP contributed to its excellent emulsification properties and microencapsulation ability (encapsulation efficiency > 97%). Nevertheless, the redispersing properties of microcapsules prepared with the modified RP as a wall material were significantly weaker than those of sodium caseinate. These findings provide new guidance and insights into the modulation of functional properties and applications of RP.

## 1. Introduction

Protein is an important macronutrient for humans, and a daily intake of 0.66–0.88 g/kg body weight is required to meet the body’s needs. This figure needs to be 1.0–1.5 g/kg body weight/day for pregnant women, elderly people over the age of 60 years, and children under the age of 1 year [1]. With the global population expected to reach 9.5 billion by 2050, this means that protein requirements will increase by 30–50% [2]. Traditionally, dietary proteins have been provided mainly by animal agriculture (mainly meat, eggs, and milk). However, as the production of these animal proteins is largely dependent on feeding plant-based feeds, the conversion of plant proteins into these animal proteins is only about 3% efficient, and it also results in more greenhouse gas emissions and consumes more water resources and land area [3]. Moreover, human health concerns, animal welfare, and religion are also included [4]. The combination of these factors has led to an increasing demand for the consumption of plant proteins in recent years. Therefore, the discovery and utilization of plant proteins as an alternative to traditional animal proteins is an urgent need and an important challenge that is currently being faced. Compared to legume proteins, oilseed proteins, and most cereal proteins, represented by soya, pea, and wheat, rice proteins (RPs) have the unique advantage of being hypoallergenic, but also have the drawback of being very poorly soluble in neutral aqueous solutions [5,6].

For decades, spray-dried microencapsulation technology has been widely used by the food industry for the encapsulation of a wide range of food ingredients, such as flavors, antioxidants, colors, probiotics, polyunsaturated fatty acids, enzymes, and vitamins [7,8]. For this technology, the key to controlling the quality of microencapsulated products (e.g., encapsulation efficiency, stability of encapsulated ingredients, and redispersibility of capsules) lies in the selection of wall materials [9]. Proteins are commonly used as microencapsulation wall materials due to their biocompatibility, biodegradability, and nutritional value, which are mainly dependent on the solubility and emulsification of proteins [8]. Traditionally, whey protein, sodium caseinate, and gelatin are preferred for microencapsulation wall materials [10]. In recent years, consumer demand has increasingly moved toward plant proteins, driven by the aforementioned concept of sustainability. Since spray-drying processes in the food industry almost exclusively use aqueous ingredient formulations, wall materials are required to be soluble in water at relatively high concentrations (e.g., 5–15% *w*/*v*) [11]. This leads to an important challenge in utilizing plant proteins as wall materials for microencapsulation, i.e., the generally low water solubility of plant proteins. As a result, apart from legume proteins, which have been reported to be used as microencapsulated wall materials [9,10,12], amongst cereal proteins only barley proteins have shown the same level of performance [13]. For RP, which is dominated by glutelin (about 80% dry basis), it is only soluble in aqueous solutions with pH greater than 10 and less than 3, and almost insoluble in neutral aqueous solutions [14]. Thus, enhancing the water solubility of RPs is the first prerequisite for developing them as spray-dried microencapsulated wall materials.

In response to this shortcoming of rice proteins, several strategies have been used in the last decades to improve the solubility of rice proteins in neutral aqueous solutions, mainly including enzymatic hydrolysis and chemical and physical methods [15,16,17,18,19,20,21,22]. In comparison, alkaline pH-shifted treatments exhibited the highest protein solubility results (around 3% *w*/*v*) due to their ability to induce the formation of a molten globular conformation and deamidation of its structure [18,20,23,24,25,26,27]. If the treatment temperature of rice proteins within alkaline aqueous solutions was increased, the efficiency of the improvement of protein solubility could be further enhanced [28,29]. Based on this, we recently found that RP (10% *w*/*v*) directly dispersed in sodium hydroxide aqueous solution (0.075–0.125 M) was able to be induced to form a stable gel state at room temperature. However, longer (>20 min) heat treatments (>50 °C) lead to depolymerization of RP aggregates and degradation of glutelin subunits, and the gel exhibits liquefaction [30]. Thus, an interesting scientific hypothesis is that the use of moderate heat treatments containing sodium hydroxide aqueous solutions with high RP content may provide a more applicable solution for the preparation of RP with high solubility within neutral aqueous solutions.

Therefore, in this work, the first focus is to investigate the effect of a continuous heat treatment at 80 °C on the physicochemical properties, especially solubility, of RP (10% *w*/*v*) in neutral aqueous solution within sodium hydroxide solution (C_NaOH_ = 0.075–0.125 M) [30]. Following this, the performance of treated RP as a wall material for spray-dried microcapsules is comprehensively evaluated. These findings may provide a valuable reference to promote the wide application of RP as a food ingredient.

## 2. Material and Methods

### 2.1. Materials

Rice protein (RP; about 80% protein on dry basis, as determined by the Kjeldahl method) was purchased from Anhui Shunxin Shengyuan BioFood Co., Ltd. (Chuzhou, Anhui, China). Sodium hydroxide and rhodamine B (purity ≥ 95.0%) were purchased from Shanghai Aladdin Biochemical Technology Co., Ltd. (Shanghai, China). Further, 8-anilinonaphthalene-sulfonic acid (ANS) and 5,5-dithiobis-(2-nitrobenzoic acid) (DTNB) were purchased from Sigma Chemical Co. (St. Louis, MO, USA). All other reagents were analytical grade unless otherwise stated. The water used in the experiments was purified by a Milli-Q apparatus (Millipore, Bedford, UK), with an electrical resistivity not less than 18.2 MΩ cm.

### 2.2. Heat Treatment of RP

The initial dispersion was obtained by dispersing the RP powder in deionized water under continuous stirring for 4 h at room temperature. Then, 1 M sodium hydroxide was added to the dispersion (100 mL) under continuous stirring conditions to make the final alkali concentration of the protein dispersion (10% *w*/*v*) 0.075 M (pH 11.6), 0.1 M (pH 12.4), and 0.125 M (pH 13.4). After that, the dispersion was processed in a water bath with the temperature set at 80 °C for different times (1 h, 2 h, 3 h, and 4 h) and stirring was maintained. At the end of the heat treatment, the protein dispersion was rapidly cooled to room temperature in an ice-water bath, and then the pH was adjusted to 7.0 ± 0.4. Following this, the dispersion was centrifuged at 8000 g for 30 min to collect the supernatant.

### 2.3. Determination of Solubility

The protein content of the supernatant, as described in Section 2.2, was determined using the Kjeldahl method. Protein solubility was then expressed as a percentage of the initial protein dispersion in the supernatant.

### 2.4. Measurement of Average Particle Size and Zeta Potential

The supernatant described in Section 2.2 was diluted to 1 mg/mL using phosphate buffer (10 mM, pH 7.4), and then the pH of these samples was adjusted to 3~9 by utilizing 0.1 M hydrochloric acid and 0.1 M sodium hydroxide. After that, the average particle size (including polydispersity index (PDI)) and zeta potential were determined using a Zetasizer Nano ZS90 (Malvern Instruments, Worcestershire, UK) configured with a 4 mW He-Ne laser (633 nm wavelength). The results were calculated by the Stokes–Einstein model and Henry’s equation, respectively, using the software supplied with the instrument.

### 2.5. Determination of Surface Hydrophobicity

The surface hydrophobicity of the samples was determined using an ANS fluorescent probe [30]. Briefly, the protein concentration was diluted to 0.01, 0.05, 0.10, 0.20, and 0.50 mg/mL by phosphate buffer (10 mM, pH 7.4). Subsequently, the ANS solution (40 μL, prepared with 10 mol/L pH 7.4 PBS to 8 mmol/L solution) was mixed with the diluted protein solution (4 mL), and then the fluorescence intensity of the samples was measured using a fluorescence spectrophotometer (F-4600, Hitachi, Tokyo, Japan). The excitation and emission wavelengths were set at 365 nm and 484 nm, respectively, and the slit was 5 nm. The fluorescence intensity obtained was plotted against the protein concentration, and the slope was taken as the surface hydrophobicity index of the protein molecules.

### 2.6. Determination of Free Sulfhydryl Content

The free sulfhydryl content of the samples was determined using Ellman’s reagent (5,50-dithiobis-(2-nitrobenzoic acid) (DTNB)) [30]. The DTNB reagent (50 μL, 5 mg/mL) was mixed with the protein solution (5 mL) and left to react at room temperature for 15 min, and then the absorbance was measured at 412 nm using a UV-Vis spectrophotometer (UV-3900, Hitachi, Tokyo, Japan). The free sulfhydryl content, in μmol SH/g protein, of the protein was calculated using the extinction coefficient of 13,600 M^−1^cm^−1^.

### 2.7. Determination of SDS-PAGE Electrophoresis

The above supernatant (160 μL), as described in Section 2.2, was mixed with a Tris-HCl buffer solution (10 mM, consisting of 1% dithiothreitol, 4% SDS, 2 M thiourea, 6 M urea, 20% glycerol, and 0.02% (*w*/*w*) bromophenol blue, 40 μL) and heated at 100 °C for 5 min, then centrifuged at 4 °C for 5 min at 10,000× *g*. After that, 15 μL of the supernatant was loaded onto the concentrated gel (5%) at a current of 20 mA. The current was adjusted to 40 mA after entering the separator gel (12%). After electrophoresis, the gels were stained with Komas Brilliant Blue R-250 staining solution (45% methanol, 10% acetic acid, and 0.1% Komas Brilliant Blue R-250) for 1 h and then treated with decolorization solution (methanol:acetic acid staining:water = 1:1:8, *V*/*V*/*V*) for 24 h. The gels were then placed on a gel imaging system for photographic processing [30].

### 2.8. Determination of Raman Spectra

Briefly, the samples were placed in a quartz cuvette with an optical path of 1 cm and measured by a LabRAM HR Evolution Raman Spectrometer (Horiba jobin Yvon S.A.S., Palaiseau, France). The measurement conditions were as follows: excitation wavelength 532 nm, measurement temperature 25 °C, 1 cm^−1^ resolution, 10 s exposure time, and 20 scans. The averaged spectral data from the scans of samples in the Raman spectrophotometer were baseline-corrected and normalized against the phenylalanine band at 1003 cm^−1^ [29].

### 2.9. Preparation and Characterization of Microcapsules

#### 2.9.1. Preparation and Characterization of Rice-Protein-Stabilized O/W Emulsions

The protein content of the supernatant of all samples described in Section 2.2 was first diluted to 4% with deionized water, and then primary emulsion was prepared by mixing rice protein (4%, 95 mL) with soybean oil (5 mL) using a high-speed homogenizer (XHF-DY, Ningbo Scientz Biotechnology Co., Ltd., Ningbo, China) at 8000 r/min for 1 min. Following this, the emulsion was prepared by cycling twice under 200 bar pressure using a high-pressure homogenizer (PT-10, Zhongke Pite (Hangzhou) Nanotechnology Co., Ltd., Hangzhou, China).

Confocal laser scanning microscope observation: Soybean oil and rice protein were stained with fluorescent dyes Nile red and Nile blue, respectively, and then the samples were imaged by a confocal laser microscope (LSM900, Zeiss, Jena, Germany) at excitation wavelengths of 488 and 633 nm and photographed for recording. An oil microscope with a magnification of 63× was selected for imaging observation.

Cryo-scanning electron microscopy observation: The emulsion microstructure was observed using a cold-field emission scanning electron microscope (Thermo Fisher Helios G4 UC, Dongguan, China). First, the stage loaded with the emulsion samples was submerged in liquid nitrogen for about 60 s to ensure that the samples were sufficiently frozen. Then, the frozen samples were broken to expose the cross-sectional area, sublimated at −90 °C for 10 min, and plated with gold spray for 60 s and then observed. During the experiment, the accelerating voltage was 5 kV, and the magnification was 10,000 times.

#### 2.9.2. Preparation of Spray-Dried Microcapsules

The preparation of microcapsules in powder state was accomplished by utilizing a spray dryer (BILON-6000Y, Shanghai Bilon Instrument Manufacturing Co., Ltd., Shanghai, China). The above emulsions were pumped into the spray dryer at a rate of 10 mL/min, with the inlet air stabilized at 185 °C. In addition, the outlet air temperature was set at 100 °C with a total solids content of 4%.

#### 2.9.3. Measurement of Encapsulation Efficiency

We accurately weighed 2 g of microcapsule powder in a dry triangular flask, added 40 mL of petroleum ether 3 times, each time after 2 min of shaking and filtration, and then combined the filtrate into a round-bottomed flask that had been dried to a constant weight. Then, the solvent of the filtrate was removed by a vacuum rotary evaporator at 60 °C, and the mass of the round-bottomed flask was weighed after the mass was constant. The increase in the mass of the round-bottomed flask was obtained as the surface oil mass of the microcapsules (*m*_1_). The method of determining the total oil quality (*m*_2_) of microcapsules was similar to the method of determining the surface oil quality, but the difference was that the microcapsules were treated with low-frequency and high-energy ultrasound (JY98-IIIDN ultrasonic cell crusher, Ningbo Scientz Biotechnology Co., Ltd., Ningbo, China) for 15 min (1200 W, 70% amplitude) before filtration to destroy the structure of the microcapsules, so that all of the encapsulated oils were released and dissolved in the petroleum ether, and the encapsulation efficiency (EE, %) was calculated using the following formula:

EE = (*m*_2_ − *m*_1_)/*m*_2_ × 100

where *m*_1_ is the surface oil mass of the microcapsule, g, and *m*_2_ is the total oil mass of the microcapsule, g.

#### 2.9.4. Microstructural Observation of Microcapsules

The microstructure of the spray-dried powder was observed by scanning electron microscopy (TM-300, Hitachi, Tokyo, Japan). In short, the powder was dispersed directly on a sample stage, sprayed with gold, and then the microstructure was observed at a voltage of 20 kV with a magnification of 5000 times.

#### 2.9.5. Evaluation of Redispersibility of Microcapsules

The microcapsule powder obtained after spray drying was homogeneously dispersed in PBS buffer solution (pH 7.2–7.4) and magnetically stirred (500 rpm) for 2 h at 25 °C, and its final concentration was fixed at 2 mg/mL. Photographs were taken to observe the apparent morphology. In addition, the particle size of the fresh samples was determined directly using a laser particle size analyzer (BLUEWAVE S3500, McKick Instruments, Inc., Rocky Mount, NC, USA). The results were automatically calculated by the instrument software, and all measurements were performed at 25 °C and repeated three times.

### 2.10. Statistical Analysis

All measurements were performed in triplicate, and results were expressed as mean ± SD. One-way analysis of variance (ANOVA) with a 95% confidence interval was used to assess the significance of the results obtained. Statistical analysis was carried out using SPSS 19.0.

## 3. Results and Discussion

### 3.1. Solubility, Average Size, and Zeta Potential of RP

**Figure 1:**
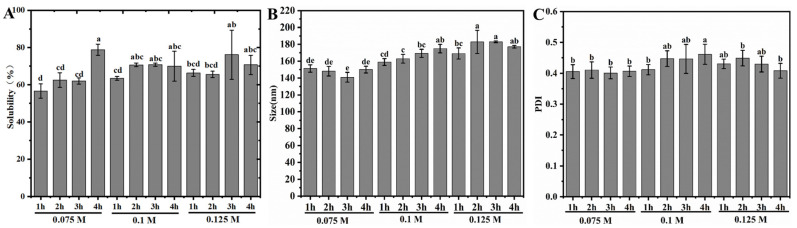
Solubility (**A**), average size (**B**), and polydispersity index (PDI) (**C**) of RP at pH 7.0 after heat treatment (80 °C) within aqueous solutions of various alkali concentrations. a–e: Different letters indicate significant differences (*p* < 0.05).

Figure 1A demonstrates the solubility of the RP in pH 7.0 ± 0.4 aqueous solution after moderate heat treatment within alkaline solution. Compared to the very low solubility of native RP in neutral aqueous solution [14], the solubility of the treated RPs all reached more than 56.3%, with the highest value approaching 81.3%. This solubility value was higher than that of the rice protein modified by enzymes (whose maximum solubility at pH 7 was slightly less than 75%, the highest level among all current reports) [31]. In addition, at a constant alkali concentration, the solubilization capacity of the RP showed an increasing trend with increasing alkali treatment time, regardless of the alkali concentration. Comparison of different alkali concentrations treated for the same time revealed that the increase in the alkali concentration did not significantly enhance the solubilization capacity of the RP. This indicated that 0.075 M alkali was sufficient to dissociate the RP in its native agglomerated state, consistent with our recent observations during alkali-induced RP gel formation at room temperature [30].

On the other hand, the average size of RP aggregates after alkali treatment was found to be in the range of 140–180 nm for all samples (Figure 1B). Since native RP in neutral aqueous solution is an irregular large-sized aggregate formed by extensive cross-linking of disulfide bonds [30], it indicates that a great degree of depolymerization effect occurs in the RP after alkaline synergistic moderate heat treatment. This is similar to our recent results using alkaline-thermal synergistic treatment on rice glutelin [28,29]. Within the alkaline solution at 25 °C, natural RP aggregates were induced to undergo depolymerization, which in turn led to the formation of a gel state through hydrophobic interactions between aggregates. However, heat treatment (80 °C) led to further degradation of rice glutelin aggregates in this gel network to a size of around 200 nm. When the heating time exceeded 30 min, both acidic and basic subunits of glutelin were degraded, resulting in liquefaction of the gel network [30]. Therefore, it can be inferred that the changes in RP solubility and average size within different alkaline solutions after moderate heat treatment should be attributed to the depolymerization of its native condensate and partial degradation of glutelin subunits. It should be noted that the PDI values of all samples were in the range of 0.4 to 0.5 (Figure 1C). This indicates that the size distribution of RP aggregates after depolymerization was not uniform enough, especially for the higher alkali concentration (0.1 M and 0.125 M) treated samples with larger PDI values than those treated with 0.075 M alkali (*p* < 0.05). This may be due to the fact that moderate heat treatment at higher alkali concentrations is more likely to lead to degradation of glutelin subunits, which, in turn, results in the formation of more aggregates of different scales.

**Figure 2:**
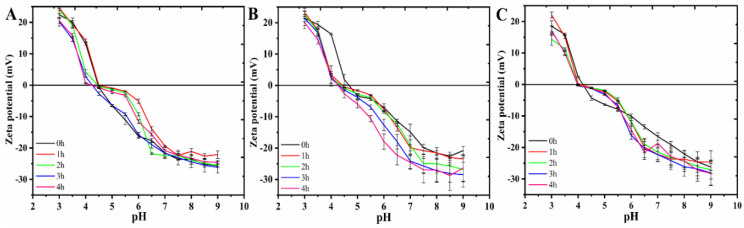
Zeta potential of RP at pH 7.0 after heat treatment (80 °C) within aqueous solutions of various alkali concentrations. (**A**) 0.075 M; (**B**) 0.1 M; (**C**) 0.125 M.

Furthermore, the effect of synergistic heat treatment with different concentrations of alkali on the stability of RP was reflected by characterizing its zeta potential, and the results are shown in Figure 2. Firstly, the profiles of the curves indicating the effect of the alkali concentration on the zeta potential of RP turned out to be similar. The zeta potentials of all samples were close to 0 in the range of pH 4.0 to 5.0, implying that this pH range is the isoelectric point region of RP. This result is consistent with the isoelectric point range of native RP [18,32]. More specifically, the zeta potential of RP after different times of treatment within 0.075 M alkali was close to that of the control samples (0 h) throughout the pH 3~10 range. However, for RP treated with 0.1 M and 0.125 M alkaline solutions, the net zeta potential in the pH 6.0~9.0 range was significantly higher than that of the control (0 h), and the longer alkaline treatment time corresponded to a higher net potential. Apparently, this change contributed to the enhancement of the aqueous solution stability of the modified RP.

### 3.2. Surface Hydrophobicity and Free Sulfhydryl Content of RP

**Figure 3:**
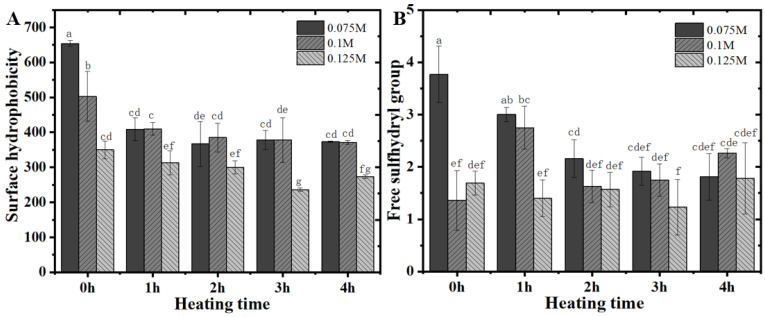
Surface hydrophobicity (**A**) and free sulfhydryl content (**B**) of RP at pH 7.0 after heat treatment (80 °C) within aqueous solutions of various alkali concentrations. a–g: Different letters indicate significant differences (*p* < 0.05).

Moreover, the surface hydrophobicity of RP also showed a decreasing trend dependent on the prolongation of the alkali treatment time, regardless of the alkali concentration (Figure 3A). This phenomenon is in the same line as our recent results on the performance of RP at pH 10–12 after alkali thermal treatment [30]. These results imply that the depolymerization induced by alkaline-thermal synergistic treatment of RP occurred such that more hydrophilic groups within the condensate were exposed [33]. It is noteworthy that the surface hydrophobicity of the control RP (0 h) exhibited an extremely significant gradient decreasing trend with the increasing alkali concentration. However, with alkali treatment from 1 to 4 h, the difference in surface hydrophobicity between all the RPs obtained from 0.075 M and 0.125 M alkali treatments was not significant, and only the samples treated with 0.125 M alkali showed a significant decrease in surface hydrophobicity. This may be due to the fact that moderate heat treatment at higher alkali concentrations is more likely to lead to depolymerization of rice glutenin subunits, thus exposing more hydrophilic regions of the protein.

On the other hand, compared to the free sulfhydryl content of native RP (about 1.8 μmol/g protein), the free sulfhydryl content of RP treated with 0.075 M alkali for 1 h at room temperature was significantly increased by about a factor of 1. The results of higher concentrations of alkali treatment were similar to those of native RP. This is similar to our recently reported results, which should be mainly attributed to the fact that the treatment with a higher concentration of alkali led to the rapid conversion of the exposed free sulfhydryl groups into disulfide bonds, which in turn led to the formation of the gel [30]. In addition, the free sulfhydryl content of the samples exhibited a similar trend of change as the heat treatment was implemented from 1 to 3 h. When the heat treatment time reached 4 h, the rice glutenin subunits were completely degraded (see the results of SDS-PAGE below), and thus no significant discrepancies were presented between the effects of different concentrations of alkali treatments.

### 3.3. Structural Characterization of RP

**Figure 4:**
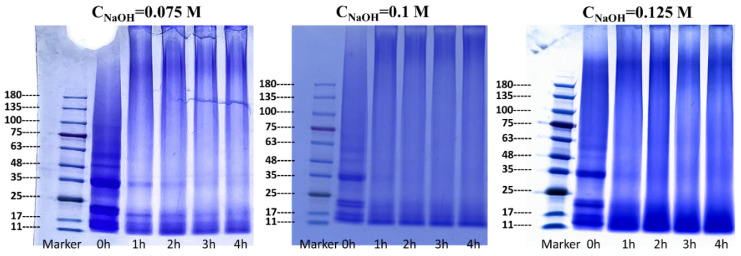
SDS-PAGE patterns of the RP at pH 7.0 after heat treatment (80 °C) within aqueous solutions of various alkali concentrations.

At first, the subunit structure of the RP was characterized using SDS-PAGE, and the results are shown in Figure 4. Regardless of the alkali concentration, the major acidic (30–40 kDa), basic (19–23 kDa), and prolamin (11–19 kDa) subunit bands in rice glutelin were clearly observed in the control [34,35]. However, with the implementation of heat treatment, the above three major subunits could also be clearly distinguished after 1–2 h of treatment within 0.075 M alkaline solution. When the heat treatment was extended to 3 h, the acidic and basic subunit bands of glutelin almost disappeared, and the band of prolamin became blurred. Similarly, the acidic and basic subunits of glutelin could not be discriminated when heat treatment within 0.1 M and 0.125 M alkaline solutions reached 1 h. These findings are consistent with our recently reported results that alkaline-thermal co-treatment led to degradation of rice glutelin subunits [30]. It was these transformations in the primary structure of rice glutelin that led to the steep increase in solubility and the formation of stable colloidal particles of RP described above. It is important to note that the smaller peptides and fragments formed by subunit degradation may also contribute to the enhancement of the emulsification of the RP after alkali treatment, mainly as a result of the easier diffusion of these hydrolysates to the interface due to their smaller size.

**Figure 5:**
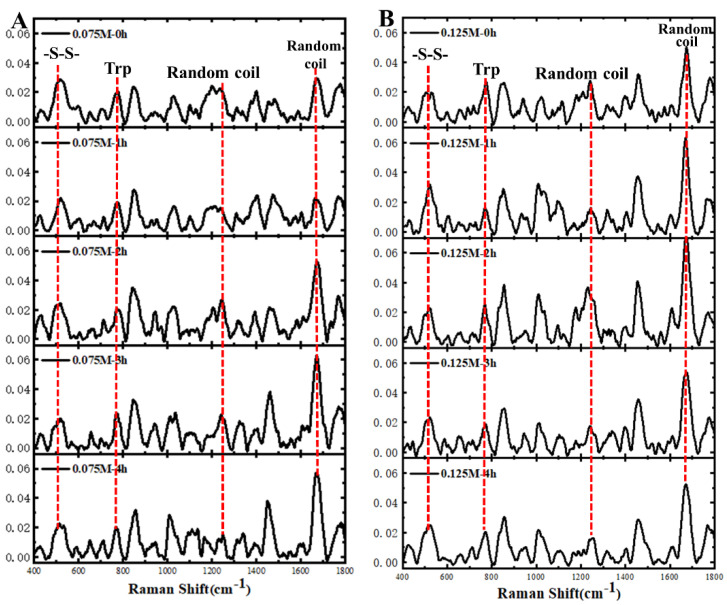
Raman spectra of the RP at pH 7.0 after heat treatment (80 °C) within aqueous solutions of various alkali concentrations. (**A**) 0.075 M; (**B**) 0.125 M.

Subsequently, Raman spectroscopy was further used to characterize the molecular backbone conformation and identity groups of RPs (Figure 5). Apparently, the characteristic peaks reflecting the disulfide bond conformation of the protein were exhibited in the 500~550 cm^−1^ region of the spectral curves of all the samples [36], a result that corresponded to the free sulfhydryl content of the RPs described above (Figure 3B). In general, the stretching vibration of the protein disulfide bond was located in the 500–550 cm^−1^ region of the Raman spectrum. The gauche-gauche-gauche (g-g-g), trans-gauche-gauche (t-g-g), and trans-gauche-trans (t-g-t) conformations of disulfide bonds were assigned to the following spectral ranges: 500–512, 513–524, and 525–540 cm^−1^, respectively [29]. Apparently, most of the samples showed characteristic peaks in the 513–524 cm^−1^ region, reflecting the conformation of the t-g-g disulfide bond. In addition, the trans-gauche-trans (t-g-t) conformation was observed for the RP treated with 0.075 M alkali for 4 h and for all samples treated with 0.1 M and 0.125 M alkali. Besides, the spectral curves of all samples showed a sharp-intensity tryptophan band at 760 cm^−1^, the presence of which implies that the indole ring residues of tryptophan were encapsulated [37]. In comparison with the intensity of native RP [29], the intensity of all samples in Figure 5 was significantly enhanced, corresponding to a weakened hydrophobic microenvironment. Furthermore, for RP treated by 0.075 M alkali, the intensity of the spectra of the amide I bands (1650–1660 cm^−1^) reflecting the protein random coil structure gradually increased with the increase in the duration of alkali treatment. On the contrary, the intensity of the random coil conformation in the amide III band (1260~1300 cm^−1^) showed a slight decrease dependent on the increase of the alkali treatment time [38]. Similarly, the RP treated by 0.125 M alkali decreased the random coil conformation in amide III, whereas this secondary structure in the amide I band showed an increasing and then decreasing trend. These results are in general agreement with our recent findings in a report on RP base-induced gel liquefaction due to heat treatment [30]. Indeed, the increase in random coil content may be closely related to the enhancement of the conformational flexibility of RP, which contributes to the conformational unfolding and rearrangement of proteins at the oil–water interface, leading to the enhancement of their emulsifying properties [29].

### 3.4. Microstructure and Physicochemical Properties of Microcapsules

#### 3.4.1. Microstructure of RP-Stabilized Oil-in-Water Emulsions

**Figure 6:**
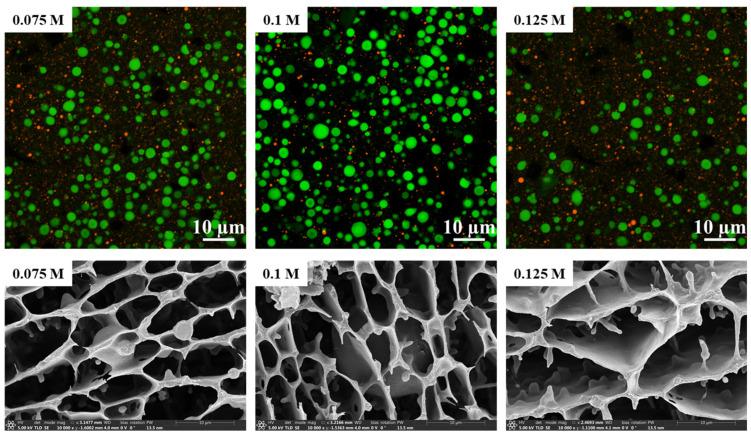
CLSM (upper row) and Cryo-SEM (bottom row) images of RP-stabilized emulsion. RP was prepared by moderate heat treatment for 1 h within aqueous solutions of different alkali concentrations.

Based on the above results of the characterization of physicochemical properties of RP after moderate heat treatment within different concentrations of alkaline solution, and with the consideration of the value of subsequent applications, herein, RP treated with 0.075 M, 0.1 M, and 0.125 M alkaline solution for 1 h was selected to evaluate its performance as a microencapsulated wall material. Initially, CLSM imaging results of RPs obtained regardless of alkali concentration treatment showed that their stable emulsion droplet (bright green and bright red corresponded to oil droplets and RP aggregates, respectively) sizes were almost always distributed in the range of 1~5 μm (Figure 6). In addition, Cryo-SEM observations further corroborated this result. However, larger-sized oil droplets than the other two emulsion samples could be observed within the RP-stabilized emulsion obtained by 0.075 M alkaline treatment. In addition to this, it was also observed that the surface of the network structure constructed by RP within all emulsion samples was very smooth, and that the network structure within the emulsions exhibited similar mesh size and morphology. These observations suggested that the RPs obtained from the three different alkali treatments had the same emulsifying ability.

#### 3.4.2. Microstructure and Encapsulation Efficiency of Microcapsules

**Figure 7:**
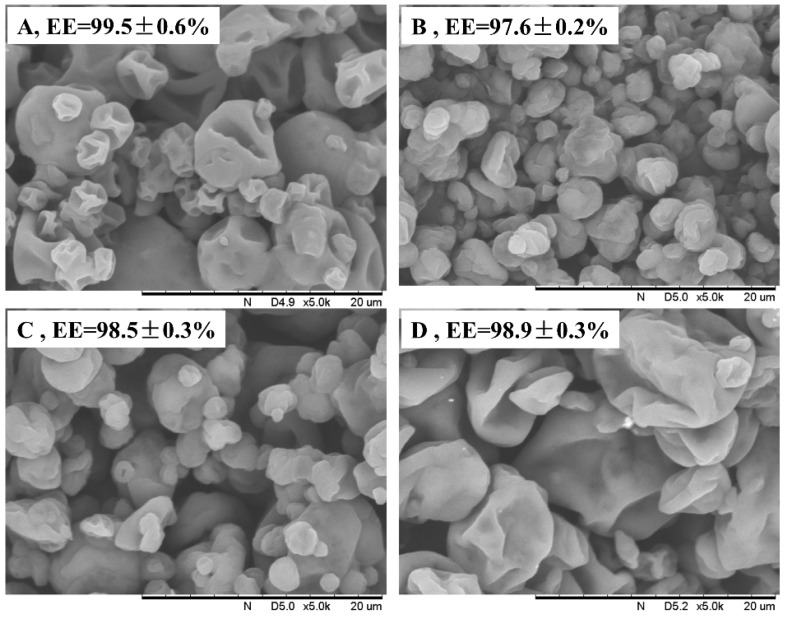
Morphology of spray-dried microcapsules. (**A**–**D**) correspond to microcapsules prepared with sodium caseinate and RP prepared by moderate heat treatment within 0.075 M, 0.1 M, and 0.125 M alkaline solution for 1 h, respectively, as wall materials.

The microstructures of the microencapsulated powders obtained from the above emulsions by spray-drying treatment are displayed in Figure 7. The surfaces of all the samples showed a pronounced indentation and wrinkled morphology, which is similar to that of the microencapsulations prepared from soybean isolate and pea protein concentrate as wall materials [10,12,39]. The appearance of this phenomenon is mainly attributed to inhomogeneous shrinkage during drying and/or cooling, associated with rapid water loss and shrinkage during the initial stages of drying. It is noteworthy that the microcapsules obtained using RP obtained after 0.075 M and 0.1 M alkali treatments were apparently less wrinkled and had a higher surface roughness of the particles when compared to RP obtained by treating with casein and 0.125 M alkali for 1 h. This result should be mainly due to the discrepancy in the rigidity of the interfacial proteins of the emulsions, with the higher structural flexibility of the sodium caseinate and 0.125 M alkali-treated RP allowing the interfacial proteins to unfold more readily and form a denser interfacial film. In addition, no obvious cracks and pores could be observed on the surfaces of all microencapsulated particles. This suggests that the particles had excellent encapsulation properties, and this conclusion was further confirmed by the results that the encapsulation efficiencies were all above 97%.

On the other hand, the size distribution of all powders was apparently very inhomogeneous, ranging from 2 to 18 μm. In particular, it should be noted that the higher concentration of RP obtained by alkali treatment corresponded to a more inhomogeneous particle size distribution. However, the degree of agglomeration between particles did exhibit the opposite trend, i.e., the lowest degree of agglomeration between microcapsules prepared with RP obtained by 0.125 M alkali treatment (Figure 7D), similar to that of sodium caseinate (Figure 7A). This variability of results may also be mainly due to the difference in the depolymerization ability of different concentrations of alkali on the rigid globular structure of rice glutelin. The above SDS-PAGE and Raman results showed that the higher alkali concentration corresponded to a higher degree of glutelin subunit degradation and random coil content. Thus, it contributed to the adsorption of proteins to the oil–water interface and the formation of a stable adsorption layer.

#### 3.4.3. Redispersing Properties of Microcapsules

**Figure 8:**
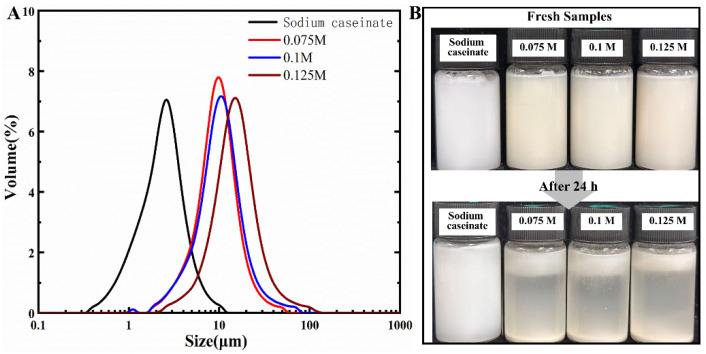
Average particle size distribution curves of spray-dried microcapsules (**A**). Morphology of microencapsulated powder redispersed into aqueous solution (**B**).

The stability of microcapsule powders prepared with different RPs as wall materials dispersed in aqueous solution was further investigated, and sodium caseinate with the same protein content was used as a control (Figure 8). The particle size distribution of the microcapsule powders prepared with sodium caseinate as the wall material dispersed in aqueous solution was in the range of 0.3–10 μm, which was significantly different from the above SEM observations, and exactly illustrated the excellent aqueous dispersion property of the microcapsule powders. In contrast, the particle size distribution of the RP-prepared powder obtained after treatment with 0.125 M alkali was in the range of 2–100 μm after redispersion, and this value was significantly higher than that observed by SEM. The reason for this may be due to the partial rupture of the microencapsulated powder occurring due to water absorption and swelling, and the same phenomenon can be observed in the RP after treatment with two other concentrations of alkali. This conclusion was further corroborated by the average size of the samples, both for the D_50_ and the D_4,3_ and D_3,2_ values, and all the average sizes of the microcapsules prepared in RP exhibited a trend consistent with the SEM observations (Table 1). In addition, the fresh samples after redispersion did not exhibit visible instability after standing at room temperature for 1 h (Figure 8B). However, all RP microencapsulated powder redispersions showed not only a clear emulsion layer after 24 h of continuous standing, but also a clear precipitation of protein aggregates at the bottom of the bottles, whereas sodium caseinate microencapsulated dispersions were still as stable as the fresh samples. The poor redispersibility of modified RP compared to sodium caseinate may be related to its thermal stability, as both have thermal denaturation temperatures of about 85 °C and 120 °C, respectively. Furthermore, the redispersion performance of these RP powders suggests that their use as microencapsulation wall materials is not yet sufficient to replace sodium caseinate, and that protein blends in the appropriate ratio may have better application value [7].

## 4. Conclusions

Dispersion of the RP in alkaline aqueous solution followed by moderate heat treatment increased the solubilization of natural RP, which is virtually insoluble in neutral aqueous solution, to more than 56.3%. This remarkable shift in the RP solubility capacity was mainly dependent on the depolymerization of rice glutenin by the synergistic effect of alkali and heat treatment, resulting in a decrease in the average protein size to 140–180 nm, as well as a decrease in the surface hydrophobicity and free sulfhydryl content of the RP. In addition, higher alkali concentrations and longer heat treatments corresponded to a higher degree of subunit degradation of rice glutelin and induced the formation of more random coil conformations of the protein. Thus, the high solubilization capacity conferred excellent emulsion stability and spray-dried microencapsulation efficiency (>97%) to RP. Nevertheless, in comparison with sodium caseinate, microencapsulated powders prepared with RP obtained by alkaline and moderate heat treatments exhibited a larger average size in aqueous solution (D_4,3_ > 10 μm) and showed significant instability after 24 h of room-temperature standing via visible separation. These findings suggested that although alkaline-thermal synergistic treatment would greatly improve the solubility of RP, its microencapsulation properties need to be further tuned. However, utilizing modified RP to partially replace sodium caseinate is an option with practical value in terms of economic cost and nutritional aspects.

## Figures and Tables

**Table 1:** Average size of microencapsulated powders prepared with different wall materials redispersed in aqueous solution.

Type of Protein	D_50_ (μm)	D_4,3_ (μm)	D_3,2_ (μm)
Sodium caseinate	2.271	2.518	1.8
RP (0.075 M + 1 h)	9.15	10.29	7.75
RP (0.1 M + 1 h)	9.75	11.5	7.98
RP (0.125 M + 1 h)	14.19	16.98	11.92

## Data Availability

The original contributions presented in the study are included in the article. Further inquiries can be directed to the corresponding author.
